# Two Cases of Metastatic Abscesses Following Surgical Operations, in the Service of Mons. Roux, Hotel Dieu, Paris

**Published:** 1849-10

**Authors:** J. H. Weir

**Affiliations:** Philada.


*Two Cases of Metastatic Abscesses following Surgical Operations, in the service of* Mons. Roux, *Hotel Dieu, Paris.* Reported for the Medical Examiner, by J. H. Weir, M. D., Philada.

Julien, 21 years of age, received a blow on the frontal region by falling against the corner of a door. Entered Hospital St. Antoine, where he remained eight days. No symptoms of concussion or compression of the brain after the accident. The small wound cicatrized rapidly. Six days after leaving the hospital, an abscess formed at the lower part of the cicatrix, which was opened with a bistoury. Patient then entered Hotel Dieu, April 30th.

*April 30th.* Abscess situated on a level with the frontal protuberance, one inch in length, and half an inch in breadth. Suppurates freely; the bone is bare; fever intense; little appetite; ordered simple dressings; absolute diet. After the lapse of four days the fever declined, and at the expiration of the eleventh day, had ceased. The pus was of a good quality; the integuments surrounding the wound were not inflamed; appetite good.

On the night of the 7th, the dressings of the wound became detached, so that the wound was exposed to the air during six or seven hours. A short time afterwards, the patient was taken with a chill; dry heat of skin; vomiting of bilious matter; pain between the shoulders.

*May 8th.* Vomiting still continues; the patient is in a very low state; pulse small and frequent; skin hot and dry; the pain between the shoulder has augmented. On percussion, dull sound at the base of right lung; respiratory murmur absent at that point Pleurisy diagnosed with symptoms of purulent infection.

Ordered Tart. Emet. grs. iij.

*May 9th.* A severe pain felt in the region of the liver; excessive prostration. *The wound is dry.* Ordered blister to-night; hypochondrium.

*May 10th.* Same state of prostration; pulse very small; severe pains in the region of the liver; laborious cough; diarrhœa, icteric tinge of the skin.

*May 11th.* Same state. Death at 12 M.

*Autopsy.* An icteric tinge over the whole body; the wound of the forehead is enlarged, and the periosteum detached; no fracture of cranium; surface of coronal suture is rugose and bathed in pus. On examination of the internal face of the bone, it is found to be denuded; its greyish color, its rugosities, show that there has been effusion of pus from the external to the internal surface. The dura mater beneath the wound is of a blackish color, slightly softened and soaked in pus; no false membranes above the arachnoid; slight injection of the surface of the brain; the cerebral substance is of a normal consistence and color.

Pleurisy of the right side with exudation of a turbid liquid. Pseudo membrane enveloping the lower lobe of right lung;—underneath this membrane, several superficial *metastatic* abscesses; no induration of lung; some metastatic abscesses in left lung; liver healthy. In the interior of the spleen, three metastatic abscesses; one in suppuration. Stomach and intestines healthy. Jugular veins and the sinuses of the dura mater contain some black clots slightly consistent, without traces of pus.

### *Case of Stricture of the Urethra—Forced Catheterism—Purulent Infection—Death.*

In bed No. 5 is a man who entered the hospital for a stricture of the urethra, 20th November. The immediate cause of his entry was a retention of urine, which had lasted fourteen hours.

The stricture is of six years standing, having followed an attack of gonorrhœa, which had been allowed to take its own course without any treatment. The stricture is seated at the commencement of the prostatic portion of the urethra, and is excessively firm ; the patient has been accustomed to use bougies for a number of years, and it was not till he found it impossible to overcome the obstruction, that he determined to apply for surgical advice.

M. Roux made use of the conical silver catheter introduced into notice by Boyer. The first attempt was unsuccessful. A false passage existed on the right side of the urethra, apparently running into the prostate. An hour after the first trial, Mr. Roux succeeded in introducing the catheter, not without difficulty, and the employment of much force. Nearly a quart of urine flowed through the catheter, and the instrument was left *in situ* for twenty-four hours. During this time the patient suffered extremely, especially in the region of the prostrate and the *bas ventre.* Some blood flowed through the canula for a few hours. Ordered laxative injection, with an anodyne draught.

*Nov.* 21*st.* This morning the silver catheter was withdrawn, and a flexible gum catheter substituted. Patient passed a restless night ; pulse 100 ; skin dry and hot ; tongue dry ; pain severe in the prostatic portion of the urethra and around the anus. On examination, a tumor was felt in the perineum, just in front of the membranous portion of the urethra.

Treatment the same.

*Nov.* 23*d.* This morning patient seems better ; suffers less, and passed a quiet night. Febrile symptoms diminished ; pulse 80 ; pain still felt in perineum, and the tumor has somewhat increased in size ; no appearance of fluctuation. Urine continues to flow by the canula, mixed with some flocculi of pus. Ordered cataplasm to perineum.

*Nov.* 25*th.* Patient has had several chills during the night ; complains of general malaise ; expression of the countenance altered ; skin cold ; pulse quick and small ; tumor about the same size, and on examination, an obscure fluctuation may be felt in it. Urine continues to be mingled with pus.

*Nov.* 26*th.* In the same state ; some chills in the night ; the skin is cold, and tongue dry ; great thirst ; there is evident fluctuation in the tumor.

*Nov.* 28*th.* Patient is losing strength rapidly ; his countenance is much altered ; the eyes are sunken with the conjunctiva of a dingy-yellow hue ; the skin is cold, dry, and of a dirty-white color; face is *terreuse.* The tongue is dry and coated ; the pulse weak, thready and quick. Diarrhœa has set in; six stools during the day. The pain is still persistent in the tumor, in which the fluctuation is very evident.

*Nov. 30th.* Same state. Decubitus dorsalis; slight delirium during the night, in which the patient succeeded in withdrawing the sound. *Patient now complains of pain in the chest, more particularly on the right side.* On examination, nothing is found abnormal, either in the lungs or heart.

*Dec. 2d.* Patient is sinking. His pulse is almost imperceptible, with cold extremities; decubitus dorsalis; pain in the chest; continuance of pain in the perineum and *bas ventre.*

*Dec. 3d.* Death in the night.

*Autopsy.* The examination of the parts primarily affected showed that the stricture was situated at the commencement of the prostatic portion of the urethra. A false passage had been formed prior to the introduction of the conical catheter. The passage was situated to the *right* of the urethra, and between it and the prostate gland. M. Roux, in his forced catheterism, had also formed a false route, or rather gutter, for the lower surface of the urethra had been ruptured to the extent of an inch. The bladder was much thickened, and its mucous coat in some places ulcerated.

A large abscess had formed in the prostate, which communicated with another seated in the loose cellular tissue between the bladder and rectum. *The lungs were filled with metastatic abscesses. Small deposits of pus were found in the liver.* The stomach and intestines were healthy, as well as the brain and its meninges.

